# Food insecurity among active duty soldiers and their families during the coronavirus disease 2019 pandemic

**DOI:** 10.1017/S1368980022000192

**Published:** 2022-01-24

**Authors:** Matthew P Rabbitt, Matthew R Beymer, Joanna J Reagan, Brantley P Jarvis, Eren Y Watkins

**Affiliations:** 1 U.S. Department of Agriculture, Economic Research Service, PO Box 419205, MS 9999, Kansas City, MO 64141-6205, USA; 2 U.S. Army Public Health Center, Behavioral and Social Health Outcomes Program, Aberdeen Proving Ground, MD, USA; 3 U.S. Army Public Health Center, Health Promotion and Wellness Directorate, Aberdeen Proving Ground, MD, USA

**Keywords:** Coronavirus Disease 2019, Food insecurity, Food security, Military, Pandemic

## Abstract

**Objective:**

We examined the determinants of food insecurity among active duty Army households that transitioned into food insecurity during the coronavirus disease 2019 (COVID-19) pandemic.

**Design:**

We compared Army households that recently transitioned into marginal food insecurity with those households that remained highly food secure (*n* 2832) to better understand how these households differ in their resilience to food insecurity during economic downturns using data from a military installation in the USA in 2020.

**Setting:**

A US military installation in the USA.

**Participants:**

Active duty US Army soldiers.

**Results:**

Prior to the pandemic, the prevalence of marginal food insecurity among Army households was similar to that reported for households in the general population. Marginal food insecurity among Army households increased over 1·5-fold – from 19 % to 33 % – with the onset of the pandemic. Relative to Army households with consistently high food security, the Army households that transitioned into marginal food insecurity after the onset of the pandemic were more likely to report concerns about financial insecurity and the job security of their family members.

**Conclusions:**

Army households, like their civilian counterparts, are vulnerable to food insecurity because of instability in their income during periods of economic uncertainty. Periods of economic uncertainty are more common for Army households because of the frequent relocations associated with military service which could lead to predictable periodic spikes in their food insecurity.

In 2020, the coronavirus disease 2019 (COVID-19) pandemic, and implementation of public health policies to minimise the spread of infection, caused the US economy to contract. This contraction resulted in reductions in household income through the loss of employment and through reductions in hours of work for those who remained employed. In April 2020, the unemployment rate increased by 10·3 percentage points to 14·7 %, and the number of individuals working part-time for economic reasons nearly doubled^(1)^. Reductions in the total labour supply of households contributed to increases in the severity of their food insecurity^(2)^, defined as limited or uncertain access to food because of a lack of resources, during the pandemic^(3)^.

Research on changes in the severity of household food insecurity during economic downturns has focused on the relationship between income, prices, the unemployment rate, or a combination of the three, and food insecurity. With the onset of the Great Recession in 2008, the prevalence of food insecurity increased by 32 % (from 11·1 % to 14·6 %) and did not return to the pre-recessionary levels until 2018^(3)^. Year-to-year changes in food insecurity at the macroeconomic level are most often associated with changes in the unemployment rate, inflation and the relative price of food^(4)^. Restrepo, Rabbitt and Gregory^(2)^ reported that households with respondents who lost their jobs due to pandemic-induced firm closures spent less on food, were less likely to have enough food to eat and were less likely to report confidence in their ability to afford food in the future. Households that cannot adequately adjust their spending during economic downturns because of negative income shocks are particularly vulnerable to increases in their food insecurity which may be difficult to reverse^(5)^.

With the onset of the COVID-19 pandemic, food insecurity increased from 10·5 % in 2019^(3)^ to 21·9 % between late March and early April 2020^(6)^. Increases in food insufficiency – a more severe indicator of food inadequacy that measures whether a household generally has enough to eat – during the COVID-19 pandemic were strongly associated with adults who are minorities, who are living at or near the poverty line, or who live in larger households^(7)^. Moreover, between April and June 2020, nearly a quarter of households with children reported their food did not last in the prior week^(8)^, and nearly 10 % of parents reported their children were experiencing the most severe forms of food insecurity in October 2020^(9)^.

Limited research suggests that military households are vulnerable to food insecurity^(10,11)^. These households represent a sizable demographic group in the USA. For example, in 2019, there were 1 326 000 service members on active duty^(12)^. Moreover, there were more active duty family members than there were service members. There were 1 591 042 family members associated with an active duty service member in 2019, and nearly two-thirds of these family members were children^(12)^.

Active duty military households are susceptible to food insecurity because of instability in their total household income, the primary determinant of food insecurity^(13)^. Although active duty service members typically earn more than civilians with a comparable level of education and have more stable employment^(14)^, their spouses are placed at a considerable disadvantage in the civilian labour market because of the service member’s long work hours, deployment schedules and frequent relocations^(14)^. Spouses of active duty service members are more likely to be unemployed, and if they are employed, they work fewer hours per week and per year, and earn less than their civilian counterparts^(14–18)^. As a result, an active duty military household’s total household income can be at or below that of comparable civilian households. Roughly half of employed military spouses report their reason for working is to pay bills and cover basic expenses^(15)^. Given that military spouses are overwhelmingly (90·9 %) female^(12)^, and employment among females has been disproportionately affected by the pandemic^(1)^, it is highly likely that active duty military households are experiencing food insecurity during the COVID-19 pandemic.

The issue of food insecurity among active duty military households is further complicated by the fact that they utilise the US Department of Agriculture’s (USDA) food and nutrition assistance programmes at lower rates than civilian households. London and Heflin^(19)^ find that only 2·2 percent of active duty service members participated in the Supplemental Nutrition Assistance Program (SNAP, formerly the Food Stamp Program) – the largest of the USDA food and nutrition assistance programs – between 2008 and 2012. Their relatively low participation rate may be an artifact of self-selection or an indication that programme eligibility rules are creating unintended barriers to participation. Active duty military households that do not occupy government-owned housing receive an in-kind housing benefit each month in addition to their basic pay. A service member’s basic allowance for housing (BAH) is included in the income of active duty military households when determining their SNAP eligibility even though the military considers BAH an entitlement. In a recent working paper, Giombi *et al*.^(20)^ simulate changes in SNAP eligibility under different BAH exemption levels. They find that exempting a service member’s BAH from their income when determining their SNAP eligibility would increase programme eligibility among active duty military households by as much as 70 percent.

To date, research on food insecurity among households of active duty service members in the US Armed Forces is limited. To our knowledge, no studies have considered how economic downturns impact the food insecurity of military households, and there is limited evidence of the prevalence of food insecurity among these households. In this article, we examine the characteristics and determinants of food insecurity among a sample of US Army households with an active duty soldier (subsequently referred to as ‘Army households’) who transitioned into food insecurity after the onset of the pandemic.

## Methods

A Behavioral Health Epidemiological Consultation is conducted by the US Army Public Health Center’s Behavioral and Social Health Outcomes Program when there is a perceived increase in suicidal behaviour or other behavioural or social health issues by military leadership. Behavioral Health Epidemiological Consultation surveys are designed to address the specific public health needs of a military installation.

The survey for this Behavioral Health Epidemiological Consultation collected information on soldiers’ demographics, military characteristics, nutrition, mental health, social support and COVID-19 pandemic-related stressors. The data were collected in June 2020 from one division at a US Army installation. There were over 14 000 soldiers eligible to complete the survey in the division. Among those, a total of 9967 responses were received for the survey. Responses were filtered out that were not an active duty service member (e.g. civilian or contractor), identified as a survey duplicate, failed both attention filters or had a response time to the survey that was under 10 min. Of the 9967 surveys, only 5842 met the final analysis inclusion criteria (∼42 % of the total eligible population).

Of the 5842 valid responses, 5049 (86 %) responded to both questions on food insecurity. Of those 5049 responses, 2832 either remained food stable or transitioned from food security to food insecurity and were analysed in the multivariable model. The installation where the Behavioral Health Epidemiological Consultation occurred was in the USA and had a population size over 39 000 soldiers. All other details of the installation are omitted to protect the anonymity of the respondents.

### Food insecurity measure

Respondents’ household food insecurity was assessed using two questions drawn from the USDA’s Household Food Security Survey Module^(3)^. This two-question food insecurity screener^(21,22)^ is becoming more common in healthcare settings where large quantities of information are collected in a short period of time, and in surveys where respondent burden is a concern (e.g. the National Opinion Research Center’s COVID Impact Survey). The food insecurity questions considered here are among the least severe questions used to assess a household’s food insecurity. These questions capture anxiety about meeting one’s food needs and early indications of food shortages.

Food insecurity before and after the onset of the pandemic was assessed by administering two separate versions of the food insecurity screener (see the appendix for details on the questions). The respondent is asked whether they experienced any of the conditions described by the food insecurity questions in the year before the pandemic, spanning January through December 2019, immediately followed by whether any of these conditions occurred in their household in the first 6 months of the pandemic (January through June 2020). Respondents that affirmed at least one of the food insecurity questions referencing the pre-pandemic period were classified as representing a marginally food insecure household before the pandemic while those that affirmed at least one of the food insecurity questions referencing the post-pandemic period were classified as marginally food insecure after the onset of the pandemic. Marginal food insecurity is a broader measure of food insecurity that captures households that report any indications of food insecurity^(23)^. Such households are classified as having marginal, low or very low food security according to the USDA’s food security status classification system.

We also compared military estimates to civilian estimates of marginal food insecurity. Estimates of the prevalence of marginal food insecurity among civilian households before the pandemic were obtained from the 2019 Current Population Survey – Food Security Supplement administered by the Bureau of the Census. Estimates of marginal food insecurity after the onset of the pandemic were obtained using data from the third week of the COVID Impact Survey – spanning April 20 through June 8, 2020 – for households with an adult respondent between the ages of 18 and 64. We restricted our focus to civilian households with a nonelderly respondent to increase the comparability of our estimates with those for Army households based on the June 2020 survey.

### Statistical analysis

Variables were chosen a priori in that change in a household’s food insecurity is determined by its socio-economic and demographic characteristics^(13)^. Specifically, we estimate a linear probability model for the transition from high food security before the pandemic to marginal food insecurity after the onset of the pandemic. Standard errors are corrected for heteroscedasticity.

The sample for our regression analysis of the determinants of the marginal food insecurity transition is restricted to Army households that reported transitioning to marginal food insecurity or reported they were consistently highly food secure after the onset of the pandemic. Most of the soldiers’ and households’ characteristics considered here are indicator variables except for the continuous scale variables for perceived social support^(24)^, posttraumatic stress disorder (PTSD)^(25)^ and depression and anxiety^(26)^. Descriptive statistics for our sample are provided in Table 1.

**Table 1:** Characteristics of active duty army households that are associated with the transition into marginal food insecurity after the onset of the Coronavirus Disease 2019 (COVID-19) pandemic

Variables	Total		Consistently highly food secure		Recently marginally food insecure	
	Mean	sd	Mean	sd	Mean	sd
Soldiers’ characteristics						
Female	0·103	0·305	0·098	0·298	0·128	0·334
Age						
Ages 17–29	0·711	0·453	0·710	0·454	0·718	0·450
Ages 30–39	0·235	0·424	0·235	0·424	0·233	0·423
Race and ethnicity						
Black	0·117	0·321	0·107	0·310	0·162	0·369
Asian/Pacific Islander	0·048	0·214	0·041	0·199	0·079	0·270
Hispanic	0·166	0·372	0·162	0·368	0·189	0·392
Marital status						
Widowed/divorced/separated	0·081	0·273	0·076	0·265	0·103	0·305
Single	0·398	0·489	0·411	0·492	0·335	0·472
Educational attainment						
High school diploma, GED or lower	0·359	0·480	0·345	0·476	0·424	0·495
Some college or an associate’s degree	0·365	0·481	0·361	0·480	0·383	0·487
Military rank						
Private to corporal	0·443	0·497	0·431	0·495	0·499	0·501
Sergeant to staff sergeant	0·268	0·443	0·260	0·439	0·304	0·461
Sergeant first class to sergeant major	0·080	0·272	0·075	0·264	0·103	0·305
Warrant officer 1 to chief warrant officer	0·031	0·174	0·033	0·178	0·024	0·154
Social support scale score	5·655	1·318	5·686	1·316	5·508	1·318
PTSD scale score	0·911	1·697	0·760	1·554	1·629	2·117
Depression scale score	1·000	1·604	0·882	1·509	1·564	1·895
Anxiety scale score	1·166	1·713	1·041	1·631	1·759	1·956
Household characteristics						
Financial insecurity of soldier and spouse	0·040	0·196	0·029	0·169	0·089	0·285
Worried about family members’ job security	0·156	0·363	0·126	0·332	0·298	0·458
Number of child dependents	0·742	1·128	0·709	1·102	0·897	1·233
Number of active duty army households	2832		2339		493	

Means and standard deviations were estimated using unweighted data on Army households from a June 2020 Behavioral Health Epidemiological Consultation survey of soldiers at a large military installation in the USA.

## Results

**Fig. 1:**
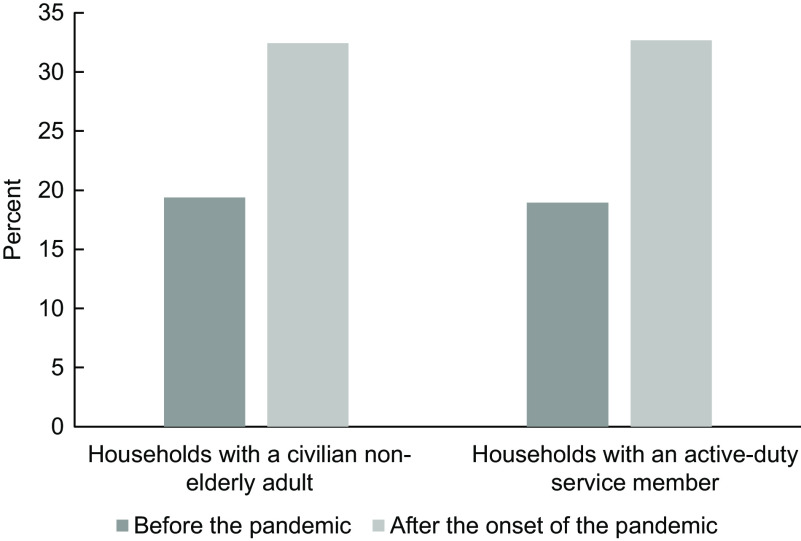
The prevalence of marginal food insecurity among samples of active duty and civilian households before and after the onset of the Coronavirus Disease 2019 (COVID-19) pandemic

The sample for this analysis was primarily male, White and junior enlisted and therefore is a similar demographic profile to the overall US Army. For more information on the demographic profiles of our sample and the US Army, see Appendix Table A.1. Figure 1 summarises food insecurity among military and civilian households before and after the onset of the pandemic.

Food insecurity among Army and civilian households increased over 1·5-fold with the onset of the pandemic (Fig. 1). Among Army households, marginal food insecurity increased from 19·0 % in the period of January through December 2019 to 32·7 % in January through June 2020. Civilian households experienced a similar increase in marginal food insecurity: from 19·4 % in January through December 2019 to 32·4 % in May through June 2020. Army and civilian households had similar rates of marginal food insecurity before and after the onset of the pandemic.

Table 2 summarises the frequency of food insecurity transitions among Army households following the onset of the pandemic. Army households reported whether they were highly food secure (i.e. showing no signs of food hardship) or marginally food insecure (i.e. showing some signs of food hardship), separately, both before and after the onset of the pandemic. Diagonal elements of the table represent Army households that reported their food insecurity did not change; the off-diagonal elements contain those who reported changes in their food insecurity.

**Table 2:** Frequency of active duty army households that reported their food insecurity changed after the onset of the Coronavirus Disease 2019 (COVID-19) pandemic at a US army installation

		After onset of the COVID-19 pandemic	
		Marginally food insecure	Highly food secure
Before COVID-19 pandemic	Marginally food insecure	825	116
	Highly food secure	788	3320

The number of Army households transitioning into marginal food insecurity after the onset of the pandemic was calculated using unweighted data from a June 2020 Behavioral Health Epidemiological Consultation survey of soldiers at a large military installation in the USA.

Nearly two-thirds (65·8 %) of Army households remained highly food secure following the onset of the pandemic. While most Army households are consistently highly food secure, approximately one in seven (15·6 %) Army households in this sample transitioned from being highly food secure before the pandemic to being marginally food insecure after the onset of the pandemic. Moreover, an additional 16·3 % of Army households in this sample reported they were consistently marginally food insecure.

Table 1 presents descriptive characteristics for Army households, calculated separately for all Army households, Army households that remained highly food secure after the onset of the pandemic, and Army households that transitioned into marginal food insecurity after the onset of the pandemic.

Army households that reported an increase in their food insecurity with the onset of the pandemic are more likely to be unmarried, racial and ethnic minorities, have lower educational attainment, reported relatively poor mental health and express concerns about their financial security and the employment stability of family members. Beyond these socio-economic characteristics, there are military characteristics that are also associated with households that reported transitioning into marginal food insecurity. Households with enlisted soldiers, particularly those in the most junior enlisted ranks, were more likely to report transitioning into marginal food insecurity after the onset of the pandemic. In addition, Army households with a soldier who screened positive for PTSD were more common among those reporting an increase in their food insecurity than those who were consistently highly food secure.

Table 3 lists estimates of the determinants of an Army household’s transition into marginal food insecurity after onset of the pandemic. Concerns about a family member’s job and the household’s financial insecurity are associated with an increased risk in food insecurity after adjustment for other covariates. Among Army households that reported concerns about a family member’s job security, there was a 14·5-percentage-point increase in the risk of reporting recent transition into marginal food insecurity after the onset of the pandemic. Households with a soldier that identifies as Black or Asian/Pacific Islander are associated with an increased risk of transitioning into marginal food insecurity after the onset of the pandemic. Additionally, a Black or Asian/Pacific Islander soldier in the household was associated with a 5·4- and 11·0-percentage-point increase, respectively, in the risk of transitioning into marginal food insecurity after the onset of the pandemic. We also find that households with children are associated with an increased risk for transitioning into marginal food insecurity.

**Table 3:** Determinants of the transition into marginal food insecurity among a sample of active duty army households after the onset of the Coronavirus Disease 2019 (COVID-19) pandemic

Variables	Recently marginally food insecure	
	Estimate	SE
Soldiers’ characteristics		
Female	0·010	0·024
Age		
Ages 17–29	0·057	0·036
Ages 30–39	0·017	0·033
Race and ethnicity		
Black	0·054**	0·025
Asian/Pacific Islander	0·110***	0·038
Hispanic	0·011	0·020
Marital status		
Widowed, divorced or separated	0·006	0·028
Single	−0·037**	0·017
Educational attainment		
High school diploma, GED or lower	−0·006	0·028
Some college or an Associate’s degree	−0·040	0·027
Military rank		
Private to corporal	0·119***	0·029
Sergeant to staff sergeant	0·105***	0·029
Sergeant first class to sergeant major	0·138***	0·038
Warrant officer 1 to chief warrant officer	0·082**	0·042
Social support scale score	0·000	0·006
PTSD scale score	0·028***	0·006
Depression scale score	0·009	0·007
Anxiety scale score	0·005	0·006
Household characteristics		
Financial insecurity of soldier and spouse	0·148***	0·045
Worried about family members’ job security	0·145***	0·023
Number of child dependents	0·025***	0·009
Constant	0·037	0·050
Sample mean of dependent variable	0·174	
*R*-squared	0·098	
Number of active duty Army households	2832	

** Significant at 0·05 level.

*** Significant at 0·01 level.

A linear probability model was estimated using unweighted data on Army households from a June 2020 Behavioral Health Epidemiological Consultation survey of soldiers at a large military installation in the USA. Standard errors are robust to heteroscedasticity.

Military rank and the severity of PTSD were also associated with an increased risk of transitioning into marginal food insecurity after the onset of the pandemic. We find that having an enlisted or warrant officer soldier in the household is associated with a greater risk for increases in the household’s food insecurity after the onset of the pandemic than having an officer in the household. Also, the risk of an Army household transitioning into marginal food insecurity is positively associated with the severity of the soldier’s screening for PTSD. Notably, being a single soldier was the only protective factor against the transition into marginal food insecurity after the onset of the pandemic.

## Discussion

In this article, we set out to examine the characteristics and determinants of households with an active duty soldier who reported transitioning into marginal food insecurity after the onset of the COVID-19 pandemic. By examining the characteristics of these Army households, we were able to identify those households that are the most vulnerable to food insecurity because of negative income shocks. Before the pandemic, nearly one in five Army households were marginally food insecure, a statistic similar to the 2019 Current Population Survey – Food Security Supplement findings from comparable civilian households. Marginal food insecurity among Army households increased over 1·5-fold with the onset of the pandemic, during which one in three of the households reportedly experienced marginal food insecurity. Ziliak^(7)^ reached a similar conclusion using the food insufficiency indicator to measure food hardship among the general population during the pandemic. However, roughly two-thirds of Army households remained highly food secure after the onset of the pandemic, which suggests most households are resilient to increases in their food insecurity during major economic downturns. Further exploration of the households that reported transitioning into marginal food insecurity after the onset of the pandemic reveals several characteristics that set them apart from households that were consistently highly food secure during this period.

Although most Army households reported no signs of food insecurity after the onset of the pandemic, approximately one in seven households transitioned into marginal food insecurity during this period. An Army household’s financial security and the job security of family members were among the primary factors associated with the household’s transition into marginal food insecurity after the onset of the pandemic. These findings speak to the importance of maintaining an Army household’s total income during periods of economic uncertainty when these households are at an increased risk of food insecurity. Notable examples of periods when Army households are likely to experience a negative shock to their total income include major economic downturns, military relocations and the deployment of a service member. Moreover, our findings demonstrate the importance of military spousal employment in ameliorating their household’s food insecurity. When surveyed, nearly half of military spouses reported they were working to pay the bills and cover basic expenses^(15)^. However, military spouses were already at a disadvantage in the civilian labour market^(14–18)^ before the pandemic, and given that military spouses are more likely to be female^(12)^, and that the pandemic’s impact on the labour market affected females disproportionately, the pandemic further exacerbated their disadvantage in the labour market. Further evidence of the importance of stable employment for military spouses is provided by the fact that the only protective factor against the transition into marginal food insecurity was being a single service member who does not rely on the contribution of other individuals to their total income.

Our findings also suggest that the likelihood of an Army household transitioning into food insecurity after the onset of the pandemic was associated with the severity of a soldier’s PTSD. Army households with a soldier experiencing PTSD may be vulnerable to food insecurity during periods of economic uncertainty because of the disruptive nature of their condition. In a study of Iraq and Afghanistan War Era veterans, Elbogen *et al.* ^(27)^ found that PTSD was associated with financial difficulties. Moreover, the stress of food insecurity during the pandemic may have also exacerbated a soldier’s PTSD. Fang, Thomsen and Nayga^(28)^ found that food insecurity was associated with a 257 % and 253 % higher risk for anxiety and depression, respectively, during the pandemic. Given a soldier’s intentions to remain in the Army are associated with their mental health and food insecurity^(10)^, the increase in food insecurity among Army households during the pandemic may have implications for the Army’s future retention and readiness.

Finally, like prior studies of civilian and military food insecurity, our findings show that a soldier’s race and ethnicity, rank and their number of children are associated with the likelihood of their household transitioning into food insecurity after the onset of pandemic. Army households headed by a soldier who identified as a racial and ethnic minority were disproportionality represented among Army households that transitioned into food insecurity after the onset of the pandemic. Using online data for American adults, Lauren *et al*.^(29)^ also found that minority-headed households were more likely to be newly food insecure during the pandemic. Our findings also show that Army households with children and those with an enlisted soldier were more likely to transition into food insecurity during the pandemic. These findings are consistent with the findings published in the US Department of Agriculture’s food security report^(3)^, which found food insecurity increased among households with children during the pandemic.

There are several limitations that should be considered when interpreting these findings. First, analyses are based on a sample of soldiers from a large installation in the USA. This sample may not be representative of the larger US Army population, or more generally, the population of active duty households. For example, the sample contains more female soldiers, more Black soldiers, and more widowed, divorced or separated soldiers than the larger US Army population but follows the distributions of age and military rank closely. Descriptive statistics for the sample and the larger US Army population are provided in Appendix Table A.1. Given this limitation, the findings should be interpreted as an upper bound for food insecurity among Army households, based on the composition of the sample, which includes persons who are younger, less educated and identify as racial and ethnic minorities. All these characteristics are factors associated with higher rates of food insecurity^(3)^. Second, we do not attempt to estimate causal relationships between an Army household’s food insecurity and its socio-economic and demographic characteristics. Therefore, findings should be interpreted as estimates of correlations between these factors. Finally, analyses are limited by our use of a two-question food insecurity screener that provides less information on a household’s food insecurity than the larger survey module used by the USDA for official food insecurity statistics. Although the screener limits our ability to examine differences in the severity of food insecurity among Army households, the analyses are based on the largest and highest quality data set to date (to our knowledge) on food insecurity among active duty Army households. These data allow us to examine the prevalence of food insecurity within this sample. To address this limitation, findings are discussed in terms of ‘marginal food insecurity’ rather than ‘food insecurity’ because marginal food insecurity captures households with marginal, low and very low food security based on the USDA’s food security status classification system.

Like the civilian population^(6)^ in the USA, the sample of soldiers in the present study experienced an increase in food insecurity after the onset of the COVID-19 pandemic. Although several risk factors were identified for transitioning into food insecurity, future studies could examine protective factors for remaining food secure during periods of economic instability. In addition, future studies could explore the reasons for the racial and ethnic disparities observed in food insecurity incidence in the military population.

## Supplementary material

For supplementary material accompanying this paper visit https://doi.org/10.1017/S1368980022000192. click here to view supplementary material
