# Supplementary material for: Food insecurity among active duty soldiers and their families during the coronavirus disease 2019 pandemic
Source: Public Health Nutr. 2022 Jan 24;25(8):2329–36. doi: 10.1017/S1368980022000192 (PMC8861550; doi:10.1017/S1368980022000192)
Supplement: Supplementary file 1 [file S1368980022000192sup001.docx]

**Appendix**

Questions used to assess the food insecurity of active duty Army households before the COVID-19 pandemic:

The following are statements that people have made about their food situation. For these statements, please tell us whether the statement was **often true**, **sometimes true**, or never true for (you/your household).

In the year before the COVID-19 pandemic (January – December 2019),

1. “We worried whether our food would run out before we got money to buy more.”
2. “The food we bought just didn’t last, and we didn’t have money to get more.”

Questions used to assess the food insecurity of active-duty Army households after the COVID-19 pandemic:

In the six months since the COVID-19 pandemic (January – June 2020),

1. “We worried whether our food would run out before we got money to buy more.”
2. “The food we bought just didn’t last, and we didn’t have money to get more.”

Affirmative responses are indicated in bold.

**Table A.1. Characteristics of Active Duty Soldiers in our Sample and the Larger U.S. Army Population**

|  | U.S. Army sample | | U.S. Army population |
| --- | --- | --- | --- |
| Characteristics | Mean | SD | Mean |
| Sex |  |  |  |
| Female | 0.103 | 0.305 | 0.153 |
| Age |  |  |  |
| Ages 17–29 | 0.711 | 0.453 | 0.658 |
| Ages 30–39 | 0.235 | 0.424 | 0.251 |
| Race and ethnicity |  |  |  |
| Black | 0.117 | 0.321 | 0.214 |
| Asian/Pacific Islander | 0.048 | 0.214 | 0.061 |
| Hispanic | 0.166 | 0.372 | 0.161 |
| Marital status |  |  |  |
| Widowed, divorced, or separated | 0.081 | 0.273 | 0.051 |
| Single | 0.398 | 0.489 | 0.421 |
| Military rank |  |  |  |
| Private to Corporal | 0.443 | 0.497 | 0.444 |
| Sergeant to Staff Sergeant | 0.268 | 0.443 | 0.258 |
| Sergeant First Class to Sergeant Major | 0.080 | 0.272 | 0.105 |
| Warrant Officer 1 to Chief Warrant Officer | 0.031 | 0.174 | 0.030 |
|  |  |  |  |
| Number of active duty soldiers | 2,832 | | 479,785 |

Note. Means and standard deviations were estimated using unweighted data on Army households from a June 2020 Behavioral Health Epidemiological Consultation survey of soldiers at a large military installation in the United States. Socioeconomic information for the larger U.S. Army population in 2019 (the most recent year available) was obtained from the U.S. Department of Defense (2020).
